# The role of electron-stimulated desorption in focused electron beam induced deposition

**DOI:** 10.3762/bjnano.4.56

**Published:** 2013-08-14

**Authors:** Willem F van Dorp, Thomas W Hansen, Jakob B Wagner, Jeff T M De Hosson

**Affiliations:** 1 Materials Science, Zernike Institute for Advanced Materials, University of Groningen, Nijenborgh 4, 9747 AG Groningen, Netherlands; 2 Center for Electron Nanoscopy, Technical University of Denmark, Fysikvej, DK-2800 Kgs Lyngby, Denmark

**Keywords:** desorption energy, focused electron beam induced processing, scanning transmission electron microscopy, temperature dependence, tungsten hexacarbonyl

## Abstract

We present the results of our study about the deposition rate of focused electron beam induced processing (FEBIP) as a function of the substrate temperature with the substrate being an electron-transparent amorphous carbon membrane. When W(CO)_6_ is used as a precursor it is observed that the growth rate is lower at higher substrate temperatures. From Arrhenius plots we calculated the activation energy for desorption, *E*_des_, of W(CO)_6_. We found an average value for *E*_des_ of 20.3 kJ or 0.21 eV, which is 2.5–3.0 times lower than literature values. This difference between estimates for *E*_des_ from FEBIP experiments compared to literature values is consistent with earlier findings by other authors. The discrepancy is attributed to electron-stimulated desorption, which is known to occur during electron irradiation. The data suggest that, of the W(CO)_6_ molecules that are affected by the electron irradiation, the majority desorbs from the surface rather than dissociates to contribute to the deposit. It is important to take this into account during FEBIP experiments, for instance when determining fundamental process parameters such as the activation energy for desorption.

## Introduction

When the electron beam in an electron microscope is focused on a sample in the presence of a precursor gas, it can be used to locally modify the sample. This process has gained increasing interest over the past ten years and is named focused electron beam induced processing (FEBIP) [1–3]. The molecules from the precursor gas (transiently) adsorb on the sample surface and dissociate into fragments when they are exposed to the electron beam. If these fragments react with the target material to form a gaseous product, the target is etched locally (focused electron beam induced etching). If on the other hand the fragments form a residue, a deposit grows on the sample surface (focused electron beam induced deposition). In either case, the sample can be modified directly with the electron beam, in principle without the use of any extra processing before or after the electron exposure. FEBIP is applied in various fields. Because electrons can be focused into narrow beams, small patterns can be defined with FEBIP. Sub-10 nm features can be written with the e-beam [4–6] and a deposition can even be carried out molecule by molecule in a transmission electron microscope [7]. FEBIP is used to repair masks for ultraviolet and extreme ultraviolet-light lithography [8] and to create, for instance, photonic devices [9], nanowires [10], tips for probe microscopy [11], and magnetic nanostructures [12–13]. FEBIP is a complex process, in which many parameters are involved. Examples are the residence times of the precursor molecules on the surface, the cross section or the reaction rate of dissociation, the local gas flux at the sample, etc. If we want to understand and model FEBIP, we need to understand how these parameters contribute to the final product.

In this paper we determined the activation energy for desorption, *E*_des_, from a FEBIP experiment. The desorption energy plays a significant role in FEBIP, since (amongst others) it determines the residence time of the precursor molecules on the surface, which in turn affects the growth rate. The activation energy for desorption can be determined from FEBIP experiments by measuring the deposition rate as a function of substrate temperature and constructing an Arrhenius plot. Christy measured *E*_des_ in a FEBIP experiment for a siloxane (tetramethyl tetraphenyl trisiloxane, DC-704 pump oil) and found that the value found from the FEBIP experiment underestimates the desorption energy by a factor of two to three compared to reference values [14–15]. Li et al. have performed the same measurement for WF_6_ [16] and found a desorption energy that was three to five times lower than expected. Li et al. explained this difference with electron-stimulated desorption. Electron-stimulated desorption is known to occur during electron irradiation, being the result of secondary electron emission. According to Madey and Yates, “electron bombardment can promote the desorption of ionic and neutral atomic and molecular species from the surface, can alter the bonding of surface species and can cause polymerization” [17]. While the latter two processes are driving forces for FEBIP, the amount of desorption from the surface may be significant during electron irradiation.

We determined the growth rate for W(CO)_6_ as a function of substrate temperature and compare the extracted energies *E*_des_ with values found in the literature.

## Results and Discussion

**Figure 1:**
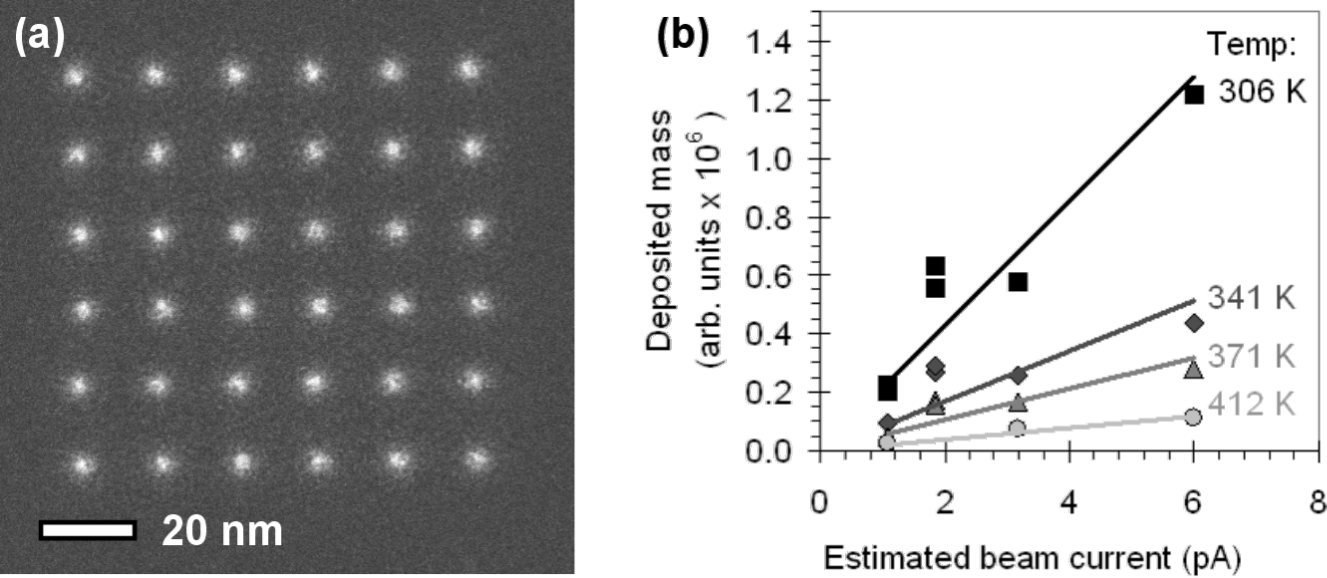
(a) An array of dots written at a substrate temperature of 306 K (33 °C) and a dwell time of 3 s per dot. (b) The deposited mass as a function of beam current and substrate temperature. The dwell time was 3 s per dot for all arrays.

Arrays of dots were written in an environmental transmission electron microscope on an electron-transparent holey carbon membrane mounted on a Au grid. The substrate temperature was varied between 306 K to 371 K (from 33 °C to 98 °C, respectively) and the irradiation times per dot were varied from 0.1 to 12 s. The precursor was W(CO)_6_ and the precursor pressure during writing was 1.7 Pa. Figure 1a shows an example of a dot array, written with an irradiation time of 6 s per dot at a substrate temperature of 341 K.

The annular dark field (ADF) signal was used for imaging. In ADF images the dot intensity is proportional to the deposited mass, assuming a constant composition of the deposited material. Therefore, the mass of each dot can be determined by integrating the ADF intensity on each position in the array. The details of this method are described in [18]. We have varied the beam current to determine whether the growth is limited by the electron flux or the precursor flux. The beam current was varied from spot 10 (low beam current) to spot 7 (high beam current). The ratio between the beam currents was measured by integrating the intensity of Ronchigrams [19] recorded on a charge coupled device (CCD) camera. The integrated intensity of the Ronchigram is proportional to the incident beam current. The beam currents were estimated by correlating the counts of the CCD camera in STEM-mode (in arbitrary units) to the reading of the fluorescent screen in TEM-mode (in A/cm^2^). The estimated beam currents are given in Table 1 and range between 1 and 6 pA, which is consistent with values reported in literature [20–21]. Figure 1b shows the average deposited mass per dot as a function of beam current and substrate temperature. In this case the dwell time was 3 s per dot for all arrays. From the fact that the deposited mass increases with the beam current, we conclude that the growth is electron-limited at all substrate temperatures.

**Table 1:** The counts on the CCD camera and the estimated beam current as a function of spot size.

spot	CCD camera counts (arb. units)	estimated beam current (pA)

10	2.1	1.1
9	3.5	1.9
8	5.9	3.2
7	11.0	6.0

**Figure 2:**
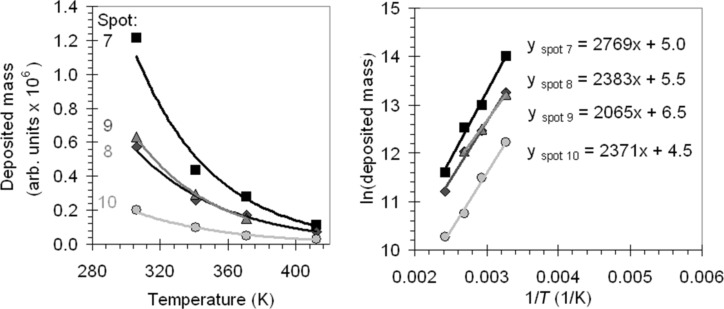
(a) The average deposited mass per dot as a function of substrate temperature and beam current. (b) The Arrhenius plot for the four beam currents, constructed from the data in (a).

The effect of the dwell time is studied by writing arrays of dots with spot 9 at three temperatures, 306 K, 341 K and 371 K. The average deposited mass per dot is plotted as a function of the dwell time in Figure 2a (see below), from which *E*_des_ can be determined. Following the model proposed by Müller et al. [22], the precursor coverage, *N*·(cm^−2^), depends on the adsorption from the gas phase, the diffusion of precursor molecules over the surface, the number of molecules consumed in the reaction with the electrons and desorption to the gas phase:

[1]
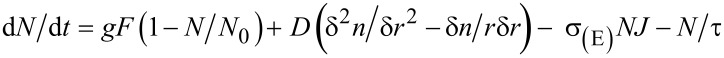


where *g* is the sticking factor, *F* is the gas flux, *N*_0_ is the density of adsorption sites in a monolayer, *D* is the diffusion coefficient, σ_(E)_ is the cross section for dissociation, *J* is the electron flux, and τ is the residence time of the molecules on the surface. The first term describes adsorption of precursor molecules from the gas phase on available sites on the substrate. The second term describes the number of molecules arriving at the writing position due to surface diffusion, the contribution of which depends on the concentration gradient. The last two terms describe dissociation by the electron beam and desorption from the substrate to the gas phase. The growth rate, *R* (cm·s^−1^), can be defined as:

[2]
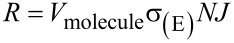


with *V*_molecule_ (cm^3^) being the volume of a deposited molecule.

Equation 1 has two temperature-dependent terms: diffusion and desorption. If, in a first approach, we assume that the supply of precursor molecules to the writing position through diffusion does not play a significant role, the effect of the substrate temperature follows simply from Equation 1. The residence time τ of the precursor molecule on the sample depends on temperature:

[3]
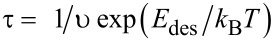


When the substrate temperature increases, the residence time of the molecules on the surface will become shorter and the desorption term in Equation 1 becomes larger. The precursor coverage, *N*, decreases, which leads to a lower growth rate *R*. In Figure 2a the same data from Figure 1b is plotted again, this time as a function of substrate temperature. The behavior described by Equation 3 is observed in Figure 1b and Figure 2a; for all beam currents the amount of deposited mass becomes smaller at higher temperatures. Based on the data in Figure 2a we made an Arrhenius plot (Figure 2b), with the natural logarithm of the average deposited mass per array as a function of 1/*T*. The slope of the fitted linear function is proportional to *E*_des_/*k*_B_. The values for *E*_des_ we calculated from the data in Figure 2b vary between 17.2 kJ/mol and 23.0 kJ/mol or 0.18 eV and 0.24 eV.

Since the dots are small (a full width at half maximum between 3 and 5 nm), surface diffusion is potentially an important precursor supply mechanism. Both desorption and diffusion are thermally activated according to the same exponential law. For an adsorbed precursor molecule the energy threshold for desorption *E*_des_ is much larger than the threshold *E*_diff_ to move from one adsorption site to an adjacent one [2]. So while molecules diffuse faster across the surface at higher temperatures, effectively the diffusion path length becomes shorter because desorption will occur sooner. This reduces the total number of precursor molecules that are transported by surface diffusion to the writing location.

**Figure 3:**
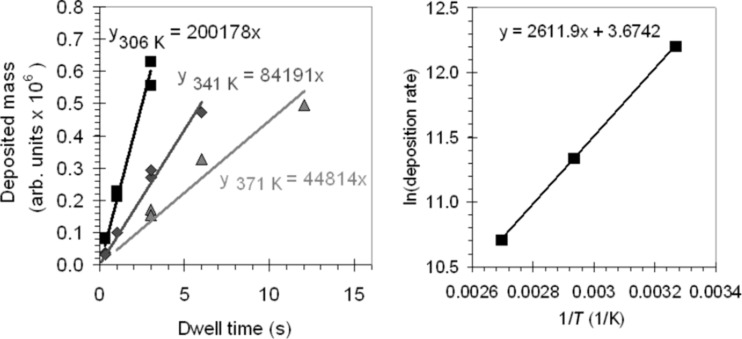
(a) The average deposited mass per dot as a function of dwell time and substrate temperature. The dots are written with spot 9. (b) The Arrhenius plot (the natural logarithm of the deposition rate as a function of the inverse of the temperature) constructed from the data in (a).

We therefore verified whether a contribution of surface diffusion is observed in the measurements. The data presented in Figure 1b (and in Figure 2a) is the result of spot exposures with a fixed dwell time of 3 s. The number of precursor molecules that arrive at the writing location through surface diffusion is time-dependent. If the contribution of the surface diffusion to the precursor transport is significant, this becomes apparent when the dwell time is varied. We deposited arrays with dwell times ranging from 0.1 s to 12.0 s as a function of the substrate temperature, all with spot 9 (i.e., a constant current density). We determine the growth rate *R* by taking the slope of the fitted linear functions in Figure 3a. In Figure 3b an Arrhenius plot is shown, where ln(*R*) is plotted as a function of 1/*T*. We found a linear dependency, from which we calculated that *E*_des_ = 21.7 kJ/mol or 0.22 eV.

**Figure 4:**
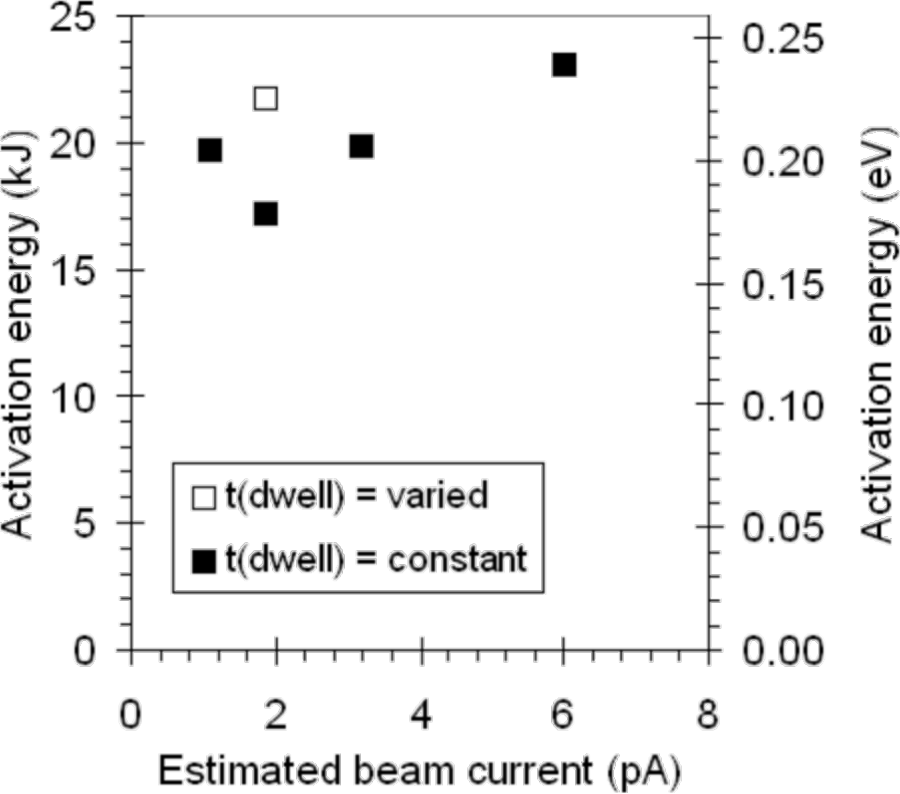
The activation energies for desorption calculated from the data in Figure 2b and Figure 3b.

The values for *E*_des_ obtained from the deposition experiments are collected in Figure 4. It is observed that the value for *E*_des_ obtained with varying dwell times (from 0.1 s to 12.0 s) falls within the scatter of the data obtained with a constant dwell time of 3 s. This suggests that surface diffusion does not play a significant role in these experiments. This finding is consistent with the value of the gas pressure. We can calculate the number of precursor molecules striking an area on the surface from the gas phase with [23]:

[4]
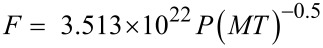


with *F* being the flux of molecules arriving at the surface, *P* the pressure in Torr, and *M* the molecular mass. A pressure of 1.7 Pa gives a flux of 1.4×10^18^ molecules·cm^−2^·s^−1^, or 1.4 × 10^4^ molecules·nm^−2^·s^−1^. Assuming a dot diameter of 4 nm and taking into account that both the upper and lower surface of the holey carbon membrane are exposed to the precursor gas, the flux on the dot area is 3.5 × 10^5^ molecules·s^−1^. In comparison, an estimated 300–1500 molecules W(CO)_6_ are necessary to form a 4 nm dot [18]. The dwell time per dot is 3 s, which makes the flux of precursor molecules arriving from the gas phase about three orders of magnitude larger than the consumption by the e-beam. Even when the sticking coefficient is smaller than 1, the transport of precursor molecules through the gas phase is sufficient to grow the dots, making the contribution from surface diffusion non-dominant in this experiment.

Figure 4 shows that the activation energy for desorption *E*_des_, calculated for W(CO)_6_, varies significantly, from 17.2 kJ/mol to 23.0 kJ/mol. Assuming a random variation, the average value for *E*_des_ is 20.3 kJ/mol (or 0.21 eV).

A reference value for W(CO)_6_ determined by temperature-programmed desorption (TPD) measurements is 53.8 kJ/mol or 0.56 eV (for desorption from multilayers of W(CO)_6_) [24]. It is observed that the values found in the FEBIP experiments are lower by a factor of 2.5–3.0. A possible explanation for this difference is the fact that the desorption energy is substrate-dependent. The FEBIP and TPD values have been determined using different substrates (amorphous carbon and Ni(100) [24], respectively). However, this does not explain the large discrepancy between the values. Measurements of *E*_des_ for MeCpPt(IV)Me_3_ (a well-known precursor for FEBIP) differ only by about 10% for the substrates Au(110) and a mixture of amorphous carbon and platinum [25]. This indicates that the factor of 2.5–3.0, which we observed here, cannot be explained solely by a substrate effect.

This conclusion is consistent with the report from Christy for a siloxane [14] and from Li et al. for WF_6_ [16]. The values for the activation energy Li et al. obtained from FEBIP experiments range from 71 meV to 210 meV, depending on the beam current (51 pA to 3400 pA) and acceleration voltage (5 to 30 kV). Similar to our findings, these values for *E*_des_ are a factor of 1.5 to 5.0 lower than the values found by TPD [26–28]. A difference to our results is that the calculated *E*_des_ does not decrease strongly with increasing beam current as Li et al. observe. However, this can be explained with a smaller range of beam currents that we use in our experiments. Li et al. varied the beam currents between 51pA and 3400 pA, in our experiments the estimated beam currents are between 1 and 6 pA.

The results demonstrate that indeed electron-stimulated desorption plays a significant role in FEBIP. The FEBIP value for *E*_des_ is three times lower than the TPD value, which we assume is more realistic. In order to find a realistic value for *E*_des_ from the FEBIP experiment, the difference between the growth rate at the lowest temperature (306 K) and the highest temperature (371 K) would need to be 25 times larger than it is in the actual experiment. In other words, of the W(CO)_6_ molecules that are affected by the electron irradiation, the majority desorbs from the surface rather than dissociates to contribute to the deposit.

This effect is not limited to WF_6_ or W(CO)_6_, but extends to electron-induced chemistry in general. According to Madey and Yates, “generally many more neutrals than ions are observed in electron-stimulated desorption” [17]. Menzel concludes that “neutrals and ions are observed to desorb under electron impact, with the neutrals contributing more than 95% of the total yield in most cases” [29]. Although the percentage of neutrals may vary with the incident electron energy [29], from our experiments it is clear that it is still significant at energies used in FEBIP. This can be important to take into account when calculating parameters such as residence times, cross sections, etc. from the amount of deposited or etched material in FEBIP experiments.

## Conclusion

The deposition rate of focused electron beam induced processing (FEBIP) has been studied as a function of the substrate temperature. Using the precursor W(CO)_6_ it was observed that the growth rate is lower at higher substrate temperatures. The measurements enables us to construct Arrhenius plots based on the measurement data, from which we calculated the activation energy for desorption, *E*_des_. We found an average value for *E*_des_ of 20.3 kJ or 0.21 eV. This is about 2.5–3.0 times lower than literature values. This difference between values measured with FEBIP and those reported in literature is consistent with findings by Christy [14] and Li et al. [16].

We contribute this discrepancy to electron-stimulated desorption, which is known to occur during electron irradiation. Electron-stimulated desorption is observed for many adsorbates and is induced by secondary electron emission. Our experimental result suggests that, of the W(CO)_6_ molecules that are affected by the electron irradiation, the majority desorbs from the surface rather than dissociates to contribute to the deposit. This is important to take into account during FEBIP experiments, for instance when determining fundamental process parameters such as the activation energy for desorption.

## Experimental

Experiments were performed on a FEI Titan 80-300 environmental scanning transmission electron microscope (STEM). A differential pumping system enabled a pressure of up to 10^3^ Pa at the sample, while keeping the rest of the column at high vacuum. The microscope was operated at 300 kV [30]. The STEM images were recorded with the annular dark field (ADF) detector at a camera length of 245 mm (inner detector angle 30 mrad). Before the deposition experiments the microscope and the sample holder were plasma cleaned. The precursor was W(CO)_6_ (CAS 14040-11-0), a low-vapor pressure solid (≈0.034 mbar at 25 °C [31]). The precursor pressure during writing was 1.7 Pa. Holey carbon membranes mounted on a Au grid were used as substrates for the FEBIP. Prior to the deposition experiments the sample was heated to 573 K (300 °C) for 45 min in the microscope at high vacuum conditions (10^−4^ Pa) in order to minimize contamination during writing.
